# Initial experiences of family caregivers of survivors of a traumatic brain injury

**DOI:** 10.4102/ajod.v4i1.165

**Published:** 2015-08-11

**Authors:** Mandi Broodryk, Chrisma Pretorius

**Affiliations:** 1 Department of Psychology, Stellenbosch University, South Africa

## Abstract

**Background:**

There seems to be a paucity of research on the initial subjective experiences of family caregivers of survivors of a traumatic brain injury (TBI).

**Objective:**

To explore the challenges that family caregivers face during the initial stages of recovery of a relative who has sustained a TBI.

**Methods:**

Thematic analysis was used to explore the findings from semi-structured interviews that were conducted with 12 female family caregivers of relatives who had sustained a TBI.

**Results:**

Family caregivers recalled their initial experiences of the shock at hearing the news about their relative’s TBI, negative experiences in hospital and frustrating interactions with healthcare professionals as particularly challenging.

**Conclusion:**

The findings of this study emphasise caregivers’ need for support, information and psycho-education, especially from healthcare professionals, from the very beginning stages of recovery from a TBI. Practical and physical needs with regard to admission to and care in the hospital were also highlighted. This research will hopefully contribute to creating awareness amongst healthcare professionals on how they can contribute to improvement of the services provided by the healthcare system based on the experiences of the caregivers who participated in this study.

## Introduction and background

Several studies have been conducted on family caregivers in the context of traumatic brain injuries (TBIs) (Arango-Lasprilla *et al.* 2010; Gan *et al.* 2010; Livingston *et al.* 2010; Phelan *et al.* 2011; Vangel, Rapport & Hanks 2011). Traumatic brain injuries (TBI) are regarded as a global public health problem, with research showing that TBIs affect an estimated 10 million people worldwide every year (Hyder *et al.* 2007). In the United States of America alone 1.7 million individuals sustain TBIs each year (Faul *et al.* 2010). An estimate of 30 000 people die due to a TBI annually and 125 000 people survive and live with disabilities as a result of TBI each year in India (Sudarsanan *et al.* 2007). In South Africa the National Health Laboratory Service (2014) reported that 89 000 new cases of TBIs are diagnosed per year.

The high number of new cases of TBIs each year in South Africa and the problems within South Africa’s healthcare system, such as shortages of hospital beds, lack of healthcare staff in the public sector, low quality of care, lack of resources for treatment or medication and high cost of institutional care (Coetzee *et al.* 2013; Department of Health 2003; George *et al.* 2012), contribute to the need for relatives to take on the caregiving role. Traditionally relatives that take on this role are generally more likely to be women (Watson 2013), and therefore most studies on caregivers tend to focus on female caregivers (Navaie-Waliser, Spriggs & Feldman 2002).

A TBI can be defined as:

a nondegenerative, noncongenital insult to the brain from an external mechanical force, possibly leading to permanent or temporary impairment of cognitive, physical, and psychosocial functions, with an associated diminished or altered state of consciousness. (Dawodu 2011:1)

The severity of TBIs is generally classified as either mild, moderate or severe, which is measured using the Glasgow Coma Scale (GCS) (Sbordone, Saul & Purisch 2007). A GCS score of 8 or below represents a severe TBI, a score of 9–12 indicates a moderate TBI, and a score of 13–15 indicates a mild TBI (Sbordone *et al.* 2007).

Individuals who sustain a TBI can present with a variety of physiological (seizures, headaches, sleep disturbances, dizziness), psychological (anxiety, depression, personality changes, psychosis) and neurobehavioural problems (retrograde and anterograde amnesia, psychiatric disorders) (Kreutzer *et al.* 2009; Trevena & Cameron 2011) that affect the relatives’ ability to function as they did before they sustained the TBI.

As a result of the consequences of sustaining a TBI the relatives will most likely need intensive rehabilitation. Rehabilitation often ranges in duration from months to a few years, and may sometimes last a lifetime (Rotondi *et al.* 2007). This may suggest that the person with the TBI will often need continued care after being discharged from hospital (Livingston *et al.* 2010), and this responsibility is then placed on relatives of these patients.

Caregivers with relatives who sustained a TBI have been found to face many challenges in the caregiving role (Arango-Lasprilla *et al.* 2010; Gan *et al.* 2010; Jumisko, Lexell & Söderberg 2007). Challenges that have been reported by relatives of patients who sustained a TBI include the impact of the physiological, psychological and neurobehavioural consequences, financial strain, insufficient time for themselves, lack of information on the consequences of sustaining a TBI, lack of understanding or empathy from others, emotional distress, stress, anxiety, depression, shock, uncertainty and a lack of resources (Braine 2011; Coco *et al.* 2011; Ergh *et al.* 2002; Gan *et al.* 2010; Jumisko *et al.* 2007; Lefebvre, Cloutier & Levert 2008; Man 2002; Marsh *et al.* 2002; McAllister 2008; Norup, Siert & Mortensen 2013; Phelan *et al.* 2011; Rotondi *et al.* 2007; Verhaeghe, Defloor & Grypdonck 2005).

Most research on this topic includes the long-term challenges that caregivers of patients with TBI face. There is therefore a paucity of research on the initial experiences of caregivers after a relative has sustained a TBI. A study was conducted on the post-traumatic stress symptoms in relatives within the first weeks of their relative acquiring a TBI (Pielmaier *et al.* 2011).However, this study did not investigate the subjective experiences of the family members within this time frame. To address this paucity of research the aim of this study was to explore the initial experiences of family caregivers after a relative sustained a TBI.

## Method

### Research question

The research question was formulated as follows: What are the challenges that family caregivers experience during the initial stages of recovery when a relative has sustained a TBI?

### Research design

An exploratory qualitative research design was used. A qualitative approach was chosen as the most appropriate methodology to explore the challenges of family caregivers of persons who have sustained a TBI, as it enabled the researcher to make an in-depth inquiry and to incorporate complex and rich insights from individuals’ personal experiences (Coenen *et al.* 2011).

### Participants

A purposefully selected sample of participants who were caring for a relative that has sustained a TBI was selected for this study. According to Crabtree and Miller (1999) five to eight participants is usually sufficient to provide rich information for qualitative research.

Participants were only included in this study if the following two inclusion criteria were met: firstly, they had to be caregivers of a person with a TBI, meaning that a fairly large amount of their time is allocated to caring for the person who has sustained a TBI; and secondly, these caregivers had to be a relative of the person who sustained a TBI.

A total of 12 participants was included in this study. As seen in Table 1, all of these participants were female and their ages ranged between 47 and 69 (mean 57) years. More than half of the participants (58.3%) reported their home language as Afrikaans. The relatives of the participants who had sustained the TBI were a daughter (58.3%), a son (25%), a husband (8.3%) or a granddaughter (8.3%). More than half of the participants were mixed-race individuals (58.3%) and the remaining participants were white. The duration of time since the relative had sustained the TBI ranged between 1 and 10 years (mean 4 years). It is important to note that participants were asked to recall their experiences of the beginning stage of their relative’s diagnosis with a TBI, and that they were not interviewed during the initial stages of TBI recovery.

**TABLE 1:** Participant characteristics.

Participant number	Age (years)	Race	Home language	Relationship to TBI survivor	Years since TBI was sustained
1	69	Mixed-race	Afrikaans	Granddaughter	4
2	59	White	English	Daughter	7
3	50	Mixed-race	Afrikaans	Daughter	2
4	64	Mixed-race	Bilingual	Daughter	1
5	56	White	Afrikaans	Son	3
6	59	White	English	Daughter	3
7	56	White	English	Daughter	10
8	65	Mixed-race	Afrikaans	Son	4
9	47	Mixed-race	Afrikaans	Son	1
10	47	White	English	Daughter	3
11	59	Mixed-race	Afrikaans	Daughter	3
12	48	Mixed-race	Afrikaans	Husband	1

TBI, traumatic brain injury. *Note:* Bilingual home language = Afrikaans and English; Relationship to TBI survivor = the capacity in which the TBI survivor is related to the caregiver. Daughter therefore refers to the caregiver’s daughter as the TBI survivor.

### Data collection

Data were collected at the Western Cape Rehabilitation Centre (WCRC) at Lentegeur Psychiatric Hospital in the Western Cape, South Africa. Potential participants were identified by the clinical psychologist from her contact with TBI patients’ relatives. They were initially contacted by the clinical psychologist from the institution and informed about this study. They were invited to take part in the study and were told that the aim of the study is to explore their initial experiences when their relative sustained a TBI. Meetings were arranged with participants who indicated that they were interested in participating in the study. Individual semi-structured interviews with 12 female caregivers were conducted; data collection was discontinued after 12 interviews, when data saturation was reached (Bowen 2008). More than half of the interviews (58.3%) were conducted at the WCRC when participants brought their relatives for check-ups or visited them in hospital, which ensured that they did not need to make additional plans for transportation. The clinical psychologist at the WCRC assisted with providing rooms to ensure that the interviews could be conducted privately in the hospital. The remaining participants were interviewed at their homes. The interviews were voice recorded for transcription purposes, with the permission of the participants.

### Data analysis

Thematic analysis was used to analyse the data. This involved the interpretation of data through identification, analysis and reporting of themes or patterns within the data set (Braun & Clarke 2006). The guidelines provided by Braun and Clarke (2006) were used by the primary researcher. First the data were transcribed whilst the primary researcher familiarised herself with them. Notes were made whilst listening to the interviews to make the coding process easier. Codes were then identified within the data set and the search for themes began. Themes and codes were reviewed and refined to ensure that nothing was left out. The software program ATLAS.ti was used to help with the data analysis process (ATLAS.ti, 7.1.3). This software was helpful in the identification of codes and translating these codes into specific themes. The last process included defining and naming of themes and subthemes. A report was written to explain and discuss the themes further and the relation between them (Braun & Clarke 2006).

### Trustworthiness

Peer examination (Krefting 1991) was implemented in this study and was pursued by discussing emergent findings at regular intervals with knowledgeable colleagues. This stimulated exploration and consideration of additional explanations and perspectives at different stages of data collection and analysis. The primary researcher used this method by discussing and comparing ideas, methods and findings with the project leader throughout the research process. Reflexivity was also implemented in this study, and necessitates carefully reflecting on the phenomenon which is being studied and also ensuring that the researcher’s own behaviour and ideologies do not affect the study (Forman *et al.* 2008; Sharts-Hopko 2002), which might have an impact on development of an accurate emic viewpoint. The primary researcher enhanced reflexivity by keeping a journal throughout the process and reflecting on it with the project leader. Emergent findings were also discussed on a regular basis with the project leader, who has extensive knowledge of TBI and experience of qualitative research.

#### Ethical considerations

Ethical approval to conduct this study was obtained from the Health Research Ethics Committee at Stellenbosch University (Ethics reference number S12/06/155). Informed consent was also obtained from each participant before data collection proceeded. It was explained to each participant that their participation was completely voluntary and that they could withdraw at any time without any negative consequences.

## Results

Three main themes that relate to the experiences of caregivers during the initial stages of their relative’s recovery emerged through data analysis. These themes were: (1) ‘shock’ at hearing the news; (2) negative experiences in hospital; and (3) frustrating interactions with healthcare professionals. All the participants reported that the accident that caused their relative to sustain a TBI was a traumatic experience for them. Nine participants reported that their relative’s TBI was caused by a motor vehicle accident; this included relatives as passengers in a motor vehicle or as a pedestrian crossing the road. Three participants reported that their relative sustained a TBI after falling; one relative fell from a moving train, one fell during a fire and another fell whilst working. Names in the participants’ extracts have been replaced with pseudonyms throughout, and coding has been used, e.g. P8 refers to participant number 8.

### ‘Shock’ at hearing the news

Most of the participants (90%) received the news of their relative’s accident either from family, friends or healthcare professionals who contacted them telephonically. This news was unexpected and involved very vague descriptions of what had happened to their relative and how serious their injuries were. One participant said she received a phone call from her son about her other son’s accident: ‘My son phoned and said, “Mommy, Brandon was in an accident. We don’t know how bad it is yet. I will phone you back’” (P8).

Another participant received text messages from several people during a meeting with a colleague, which stated that they were urgently trying to get hold of her. She phoned her one friend back: ‘So I phoned and she’s screaming and they said, “Come”. I went to the accident scene’ (P10).

The sudden nature of hearing the news of a relative who was in a serious accident left most of the participants shocked. The experience of the participants is illustrated by the following:

’See that very day of the accident was, like I said, very traumatic … So yes, that to me was the most traumatic experience of my whole life. I’ve never been in a situation like that.’ (P4)

### Negative experiences in hospital

Participants reported a number of challenges relating to their experiences at hospital when they visited their relative who had sustained a TBI. Challenges included seeing their relative in the hospital after the accident, a lack of hospital beds and the type of care received in public hospitals.

All of the family caregivers reported that their relative was in a coma after the accident occurred. They reported that it was distressing to see their relative in the hospital after the accident. One of the participants described her experience as follows: ’I was in shock, because it was just tubes and stuff‘ (P5). Another participant shared this experience:

’My son looked like a dead person and all the machines on him. It gave me a fright when I got to the hospital, to see him lying there like that‘. (P9)

Several participants (40%) reported that there was a lack of open hospital beds and their relative had to be transferred to another hospital or rehabilitation centre. The experience of helplessness is evident in the following account by participant 12:

’For some reason, the hospital transferred my husband to another hospital and there wasn’t even a bed for him. I had to take him home that night in his condition.’

Participant 5 decided to check for herself if there was in fact an open hospital bed for her son after being told by the doctor that he was being transferred. This participant reported that when she got there she was told the following: ’They told me there was no bed for him, there was no opening for my son‘ (P5).

Several participants (60%) also reported experiencing disappointment in the type of care their relative received in the hospital. One participant described her anger and frustration as follows:

’At that stage I didn’t even want to talk to them. I was very upset with them. And I am angry because his bum was burning, you get there and you tell the nurses that he pooped and then you wait and wait, you know. And then his bum was burning, so I was very angry and his genitals also burned … I told them I would take better care of him, you know’. (P5)

Similarly, another participant reported: ’She was not bathed in hospital, and when I bathed her at home she felt better‘ (P1).

Participants seem to have experienced frustration and hopelessness regarding the perceived neglect their relatives experienced in the hospital.

### Frustrating interactions with healthcare professionals

The caregivers described their initial experiences with the healthcare professionals as predominantly negative. The following extract summarises the experiences of most (80%) of the participants: ‘The biggest problem with our experience is that the medical profession do not listen… I was called neurotic, and she was called a drama queen’ (P2).

Another participant reported that her daughter was discharged from the hospital five days following her accident after sustaining a severe TBI: ‘The final words of the neurosurgeon to me were ‘’Take her home, she’ll be absolutely fine’’’ (P7).

Several participants (70%) reported that a lack of interest, support and empathy from healthcare professionals in the hospital contributed to their challenging experiences. The experiences of participants 6 and 7 emphasise the need expressed by many of the participants:

‘When we were at the hospital, to have somebody possibly who could say ‘’You know, this is my field and I’m here for you if you need me. Here’s my card, contact me’’’. (P6)

‘It would have taken a lot less of a toll on me if I had some back-up and not necessarily even just somebody to talk to, somebody to say ‘’You know what, it’s OK. This is the next step in the process you know’’’. (P7)

The participants clearly expressed a great need for guidance from the healthcare professionals about what to expect and about the recovery process.

## Discussion

Several studies have found that caring for an individual who has sustained a TBI involves many challenges (Arango-Lasprilla *et al.* 2010; Ergh *et al.* 2002; Gan *et al.* 2010; Jumisko *et al.* 2007; Lefebvre *et al.* 2008). Most studies on this topic focus on caregiving experiences from six months to several years post–injury. There have only been a few studies conducted on the initial experiences and challenges that family caregivers faced in the caregiving role. The research that is available regarding the initial stages of recovery and family caregivers’ experiences mainly include psychometric assessments of experiences and caregiver burden during this time. There is a paucity of research that focuses on caregivers’ subjective experiences in order to gain better insight into their needs and challenges during this time. It was therefore the aim of this study to examine the challenges that family caregivers face during the period during the initial stages of recovery, when a relative has sustained a TBI. It should be noted that only female family caregivers participated in this study, which is no surprise due to women in general being more likely to take on the caregiving role (Watson 2013).

The initial shock that accompanied hearing the news about the TBI of a relative was identified as a significant challenge for family caregivers. A TBI always occurs unexpectedly and suddenly (Coco *et al.* 2011). It is therefore not surprising that upon hearing the news about their relatives’ accident, family caregivers reported experiencing feelings of shock, which is commonly reported immediately after exposure to a traumatic event (American Psychological Association 2014). Furthermore, admission to the intensive care unit (ICU) and specifically coma produce strong emotions in relatives such as shock, denial, anger, despair, guilt, devastation and fear (Verhaeghe *et al.* 2005). All of the family caregivers that participated in this study reported that their relative was in a coma after the accident occurred. Seeing their relative in a coma could also have contributed to the feelings of shock, distress and devastation that they experienced. It is possible that the experiences of the caregivers can be described as a kind of ‘ambiguous loss’ as defined by Boss (Boss 2007; Boss & Couden 2002). According to Boss ambiguous loss has two dimensions: (1) a loss that relates to the physical absence but psychological presence of the relative; and (2) a loss that refers to the psychological absence but physical presence of the relative (Boss 2007; Boss & Couden 2002). The shock of hearing that a relative has suffered a TBI is an example of the second type of ambiguous loss, as the person is not dead but there is no certainty that they will ever be the same again.

According to Boss and Couden (2002):

when people are unable to obtain clarity about the status of a family member, they are often immobilized: decisions are put on hold; roles remain unclear; relationship boundaries are confusing; celebrations and rituals are cancelled. (p. 1352)

Healthcare professionals in particular should therefore be aware of the possible impact that ambiguous loss can have on family caregivers during the initial stages of TBI recovery, because their relative is often in a coma and the progress and outcome of their condition is often very uncertain. This can be an interesting avenue to be investigated more closely in future research. These speculations suggest that there is a need for support and interventions that focus on the psychological needs of the caregivers during the early stages of recovery for relatives of survivors of TBIs (Norup *et al.* 2013).

The experiences of caregivers with the hospital in general are reported to be negative. The experiences of family caregivers with healthcare providers in particular were identified as a major challenge during the initial hospital admission of their relative. Apart from the physical needs of their relatives that were not addressed (such as the availability of a hospital bed and taking care of the personal hygiene of their relative), family caregivers experienced healthcare professionals as lacking interest, support and empathy during this time. Similar to the findings of this study, Jumisko *et al.* (2007) reported that healthcare professionals often pay insufficient attention to family caregivers’ needs. There could be several reasons why healthcare professionals often do not pay sufficient attention to relatives of these patients. It might be that South Africa’s shortage of healthcare staff and therefore an increased workload makes it difficult for staff to attend to everybody’s needs (George *et al.* 2012). Dissatisfactory working conditions and low quality of care could be another reason (Coetzee *et al.* 2013). This topic could be researched further in future studies.

Whether in the form of providing information or guidance about what to expect or in the form of support to deal with their experiences of shock and trauma, family caregivers have a psychological need for support from healthcare professionals. According to Coco *et al.* (2011) it is common for relatives of survivors of a TBI to long for information from healthcare professionals. Prior research on relatives’ experiences with healthcare professionals in the ICU in general suggests that the need for accurate and comprehensible information is very important to relatives visiting their relatives in the ICU (Verhaeghe *et al.* 2005). Nurses and doctors often fail to appreciate the needs of relatives visiting their family in the ICU (Verhaeghe *et al.* 2005). It was also reported that nurses seem to underestimate their own role in satisfying the needs of relatives concerning the need for information (Verhaeghe *et al.* 2005).

## Conclusion

The findings of this study highlight the challenging experiences that family caregivers of a relative who has sustained a TBI endure from the outset and emphasise the need for support from the very beginning stages of recovery. No prior studies have investigated and reported on the early experiences of family caregivers and the challenges associated with hearing the news about their relative’s accident, diagnosis of TBI and negative experiences related to seeing their relative in the hospital. The paucity of research relating to these initial experiences might be due to the perception that family caregivers only take on the caregiving role at a later stage, when the relative with the TBI is discharged from the hospital.

In this study caregivers seem to describe their experiences as filled with shock when they first heard the news about the accident, followed by negative experiences in hospital and frustrating interactions with healthcare professionals. Interventions focusing on the provision of information and psycho-education, especially from healthcare professionals, could be beneficial to family caregivers in the initial stages of TBI recovery, as family caregivers become empowered when they gain more knowledge about their relatives’ condition (Man 2002). An increased awareness from healthcare professionals about the possible impact of ambiguous loss on the family caregivers could also contribute positively to the caregiving experience.

In conclusion, this study created a picture of the challenging experiences of caregivers during the initial stages of recovery of a relative who sustained a TBI. This research will hopefully contribute to creating awareness amongst healthcare professionals on how they can contribute to the improvement of the services provided by the healthcare system, based on the experiences of the caregivers who participated in this study.

### Limitations and future directions

Although participants reported their subjective experiences of the acute phase of TBI recovery, for most it had been more than two years post-injury. Time that has passed could affect their ability to recall their experiences accurately, and therefore it could be beneficial to conduct future qualitative research during the initial stages rather than interviewing participants about it years thereafter.

The role of healthcare professionals not only with regard to providing guidance, empathy and information to the caregivers, but also with regard to physical care (i.e. looking after the personal hygiene of their relative) was emphasised. It seems as if the caregivers view the relationship between themselves and the healthcare professionals who are involved in the treatment of their relative who sustained a TBI as very important. It was, however, evident from the findings of this study that the caregivers are generally not satisfied with the quality of the interaction between the healthcare professionals, themselves and their relatives. It might be worthwhile to explore the experiences of healthcare professionals from their perspective, in order not only to compare experiences but also to attempt to address the challenges that caregivers experience.
